# Importance of Pre-Milking Udder Hygiene to Reduce Transfer of Clostridial Spores from Teat Skin to Raw Milk

**DOI:** 10.3390/microorganisms11051337

**Published:** 2023-05-19

**Authors:** Johanna Burtscher, Tamara Rudavsky, Ulrike Zitz, Viktoria Neubauer, Konrad J. Domig

**Affiliations:** 1 Department of Food Science and Technology, Institute of Food Science, University of Natural Resources and Life Sciences, Vienna, Muthgasse 18, 1190 Vienna, Austriaulrike.zitz@boku.ac.at (U.Z.); 2 FFoQSI—Austrian Competence Centre for Feed and Food Quality, Safety & Innovation, Technopark 1D, 3430 Tulln, Austria; 3 Unit of Food Microbiology, Institute of Food Safety, Food Technology and Veterinary Public Health, University of Veterinary Medicine, 1210 Vienna, Austria; viktoria.neubauer@vetmeduni.ac.at

**Keywords:** *Clostridium*, udder hygiene, teat cleaning, milking hygiene, endospores, cheese quality

## Abstract

Butyric acid producing clostridia (BAPC) cause the so-called late-blowing defect, a serious quality problem in semi-hard and hard cheeses. Late-blown cheeses are characterized by undesired slits and cracks, irregular eyes, and off-flavors due to excessive amounts of gas and organic acids produced by clostridia. Clostridial transfer to raw milk can occur during milking through dirty teats. Therefore, teat cleaning before milking is a key factor in preventing clostridial contamination of the milk. However, different cleaning methods are used, and little information is available on the efficacy of routine teat cleaning in reducing clostridial endospores. The main objectives of this study were to assess the extent of udder contamination with BAPC spores and to investigate the efficacy of routine teat cleaning on BAPC spore counts in milk. In a longitudinal study, eight dairy farms were visited during five sampling events. Clostridial spore counts were quantified from teat skin before and after routine teat cleaning, in pooled quarter milk samples from individual cows, and in bulk tank milk samples using a most probable number method. In addition, farm management data were collected periodically through a survey, and average cow cleanliness was assessed by a veterinarian. On average, teat cleaning resulted in a 0.6 log unit reduction in BAPC spores on teat skin, and a strong positive correlation was found between BAPC spore concentrations on teat skin after cleaning and in pooled quarter milk samples. Seasonal variations and the potential influence of differences in farm management were also noted. Interestingly, average cow cleanliness correlated strongly with BAPC spore levels in milk, suggesting the potential for a quick and rough estimation method of clostridial contamination that could be implemented by farmers.

## 1. Introduction

Butyric acid producing clostridia (BAPC) cause the so-called late-blowing defect, a severe quality constraint in semi-hard and hard cheeses, resulting in a significant loss of product value and an economic burden for cheese producers [1]. Late-blown cheeses are characterized by undesirable slits and cracks, irregular eyes, and off-flavors due to excessive amounts of gas and organic acids produced by clostridia [2]. The endospores of these anaerobic bacteria are ubiquitous and common contaminants of raw milk. Their main sources in the farm environment are silage, soil, and manure [2,3]. During milking, BAPC spores are transferred to raw milk primarily via dirt consisting of feces, bedding material, and/or soil adhering to the outside of the teats [4,5]. Models of BAPC spore contamination of bulk tank raw milk led to the conclusion that the most important measure to reduce the risk of BAPC contamination of raw milk is to control BAPC spore counts in silage [6]. In addition, teat cleaning prior to milking is also essential [7,8,9].

Studies on experimental farms have shown a strong correlation between milking hygiene and BAPC spore counts in bulk tank milk. For example, Bava et al. [5] conducted a study on an experimental farm and concluded that the implementation of forestripping, teat cleaning with a detergent, and teat-dipping after milking significantly reduced the anaerobic spore count in bulk tank milk when compared to forestripping alone. By using wet reusable towels and additional dry paper towels, Magnusson et al. [10] were able to reduce the count of clostridial spores by 96% on teats that had previously been intentionally contaminated. These experimental studies provided important insights into BAPC transmission and cleaning efficacy. However, artificial contamination and standardized cleaning do not account for variations in teat microbiota, cleaning procedures, and farm management. Few studies have collected data on BAPC spores during routine teat cleaning. For example, Martin, Kent, Evanowski, Zuber Hrobuchak, and Wiedmann [8] used multi-model inference to investigate sources of bacterial spores in the farm environment that affect spore levels in farm tank milk and identified udder hygiene as one of the most important variables for BAPC spores in milk. However, the average lactating herd size in this study was 929 and may not be comparable to smaller dairy farms in Europe. As detailed knowledge of clostridial transmission to raw milk in non-experimental small farm environments is scarce, we aimed to complement and compare previously established data from experimental settings with data obtained from dairy farms during routine milking and cleaning.

The main aim of this study was to investigate the effect of routine teat cleaning and milking procedures on the number of clostridial spores in milk. To this end, clostridial spore counts were quantified on teat skin before and after routine teat cleaning, in pooled quarter milk samples from individual cows, and in bulk tank milk samples.

Secondly, with a view to developing potential mitigation and risk assessment strategies, we aimed to evaluate the correlation of clostridial spore counts in milk with an overall estimate of udder, cow, and teat hygiene. Several scoring tools have been developed to assess the level of udder and leg hygiene. Initially, these scoring tools were developed to assess the risk of mastitis. However, in previous studies, hygiene scoring according to Schreiner and Ruegg [11] showed that the presence of >40% of cows with dirty udders increased milk contamination with BAPC spores [7]. Overall cow cleanliness was also found to be better in herds with lower BAPC spore counts than in herds with higher spore counts by Nadeau et al. [12]. However, according to Cook and Reinemann [13], a more complex scoring system that takes into account the pattern of manure contamination is recommended. Therefore, we applied this adapted scoring tool to estimate the average cow cleanliness (ACC) on each farm at each sampling visit. In addition, the correlation of clostridial spore counts in bulk tank milk with routinely collected hygiene indicators—somatic cell count (SCC) and colony count (CC)—was investigated. To account for seasonal variations in clostridial spore counts, this observational study was conducted over five consecutive seasons. Variations in farm management were taken into consideration by monitoring eight farms and repeatedly collecting detailed information on farm management and hygiene measures applied on each farm.

Finally, by adding to the current knowledge of clostridial contamination of teat skin and milk in non-experimental settings, this study should shed light on potential strategies to prevent clostridial transmission to raw milk and cheese spoilage.

## 2. Materials and Methods

### 2.1. Sample Overview

**Figure 1:**
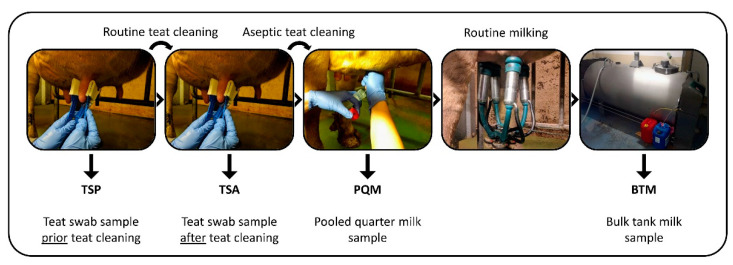
Overview of the types of samples collected on each dairy farm for the current study.

Eight Austrian dairy farms (A–H) were sampled during five consecutive seasons: summer 2018 (16 July–21 August), winter 2018 (12 November–4 December, farm C 14 January 2019), spring 2019 (18 March–2 April), summer 2019 (29 July–20 August), and winter 2019 (18 November–3 December). A 6th sampling in spring 2020 was canceled due to the COVID-19 pandemic. Each farm operated independently and had its own management style. All farmers fed silage to their dairy cows in at least two out of the five seasons. As the average number of dairy cows per farm in Austria was 18–20 in 2018 to 2019, and considering that not all cows are lactating at the same time, a representative number of 16 dairy cows per farm was sampled during each sampling event [14]. If less than 16 cows were lactating, all lactating cows were sampled (14 or 15 cows; 631 of 640 in total). The main cow breeds on the selected farms were Simmental, Holstein-Friesian, and Brown Swiss. The average milk yield per cow varied among farms and ranged from 16.6 to 31.3 kg milk. See Supplementary Table S1 for more detailed information on the characteristics of the selected farms. Samples included teat swab samples and pooled quarter milk samples. In addition, raw milk from the bulk tank of each farm was collected after the milking was finished. All samples were collected by the same veterinarian and a research assistant in the presence of the farmers during the regular milking routine (morning or evening). All samples were stored and transported at 4 °C, reaching the lab at the latest after 36 h, and stored in the lab at −20 °C until analysis. An overview of the types of samples that were collected is shown in Figure 1.

#### 2.1.1. Teat Swab samples

Teat swab samples were collected using sponge samplers moistened with 20 mL HiCap Neutralizing Broth (EZ Reach Split-Sampler, World Bioproducts LLC, Woodinville, WA, USA). One sponge sampler was used to sample two cows during the first sampling. For subsequent sampling events, the same sponge sampler was used to sample four cows. Teat samples were collected prior to cleaning after forestripping (TSP) and again after routine teat cleaning (TSA) using new sponge samplers. Each teat was rubbed twice with the front and the back of the sponge sampler. After sampling, the sponge sampler was placed in the provided bag for transport. For sample preparation, the sponge was squeezed in its bag to collect ~5 mL HiCap Neutralizing Broth in a sterile tube. The sponge sampler was then further soaked and squeezed in 15 mL buffered peptone water (Biokar diagnostics, Pantin, France), and the resulting suspension was also collected. This step was repeated once. Negative controls contained buffered peptone water only. A total of 382 (191 TSP + 191 TSA) teat swab samples were analyzed.

#### 2.1.2. Pooled Quarter Milk Samples

Before collecting a pooled quarter milk sample, the teat tips were cleaned using cotton and ethanol (70% *v*/*v*). Approximately 30 mL of pooled milk from 4 quarters from each cow (PQM) was collected in a sterile tube after routine teat cleaning. After PQM sample collection, the farmer continued with routine milking. A total of 631 PQM samples were collected.

#### 2.1.3. Bulk Tank Milk Samples

Approximately 50 mL of bulk tank milk (BTM) was collected either directly from the tank valve into a sterile tube, or the tank was opened at the top and the raw milk was collected with a disinfected ladle after thorough homogenization. One bulk tank milk sample was collected from each farm, resulting in a total of 40 BTM samples.

### 2.2. Quantification of Clostridial Spores

BAPC spore counts of all samples were determined using the AMP-6000 APS system (SY-LAB GmbH, Neupurkersdorf, Austria), which is based on a semi-automated most probable number approach as described by Brändle et al. [15]. By using a highly selective medium and 96 replicates containing 142.5 µL milk, this method allows the quantification of clostridial spores in a wide range of 73–32,000 clostridial spores per liter of milk.

#### 2.2.1. Analysis of Raw Milk Samples: PQM and BTM

Frozen samples were thawed in a water bath at room temperature. After inverting the samples 25 times according to DIN EN ISO 6887-5:2010 [16], 18 mL of milk was transferred into a pasteurization tube with a sieve insert (50 mL; 62-502200, SY-LAB). Laboratory pasteurization (20 min at 80 °C) was performed using a turbulent water bath. After the pasteurized samples were cooled to approximately 20 °C, 6 mL AmpMedia666 including additive (62-166613, SY-LAB) was added [15]. After homogenization using a vortex mixer, 96 aliquots of 190 µL of the sample–medium mixture were distributed on a 96-well plate using the pipetting unit of the AMP-6000 APS instrument. The plate was covered with a lid for incubation (48 ± 2 h at 37 °C) under anaerobic conditions (80% N_2_, 10% CO_2_, 10% H_2_) using a jar gassing system (Don Whitley Scientific Limited, Bingley, West Yorkshire, UK). Clostridial growth was indicated by a color change of the sample–medium mixture from light red to yellow. After incubation, the bottom of the 96-well plate was scanned using an AMP-6000 APS instrument and the corresponding software for qualitative sample evaluation and MPN calculation according to Hurley and Roscoe [17]. To validate the performance of the method, butyric acid producing clostridia spore standards (62-166636, SY-LAB) of known counts were periodically analyzed.

#### 2.2.2. Analysis of Teat Swab Samples and Negative Controls

The quantification of BAPC spores using AMPMedia666 as described above relies on milk as the main component providing a critical amount of nutrients. For this reason, teat swab samples, consisting of buffered peptone water, were diluted with extended shelf life (ESL) milk (3.6% fat, pasteurized and micro-filtered, milfina, Gmundner Molkerei reg.Gen.m.b.H., Gmunden, Austria). Preliminary experiments were performed to determine optimal dilutions for teat swab samples to obtain results within the method’s quantification range. A final volume of 18 mL of the diluted sample at the optimal dilution (1:18, 1:1800, 1:3600, or 1:68,400) was used for the analysis and was gently mixed with 6 mL of AmpMedia666 including additive. The following steps were performed as described above for the raw milk samples. Teat swab negative controls (sole buffered peptone water) were analyzed in the same manner as teat swab samples. The levels of BAPC spores in each batch of the diluent, the ESL milk, were checked prior to each analysis using 18 mL of milk to exclude contamination of the diluent with clostridial spores.

### 2.3. Farm Management Data, Average Cow Cleanliness, Somatic Cell Count, and Colony Count

At each sampling, each farmer completed a questionnaire about the current conditions on the farm, such as the main cow breed, type of feed, type of teat cleaning products, type of housing, and milking procedure. This data set was completed by the veterinarian with information on the average cow cleanliness based on direct observations made during sampling. ACC was scored according to Cook and Reinemann [13] using a hygiene scoring system that includes the cleanliness of three areas: (1) legs, (2) udders, and (3) flanks and upper legs of cows. The cleanliness of these areas of each cow was scored from 1 (free of dirt) to 4 (covered with baked-on dirt). After the sterile milk sampling, another PQM sample was collected from each cow in a 50 mL vial, and 2 drops of bronysolv (Analitik Austria, Vienna, Austria) were added for stabilization. Somatic cell counts (SCCs) in BTM and PQM and bacterial colony counts (CCs) of the bulk tank milk samples were determined by the respective dairies using a CombiFoss instrument (Foss, Hillerød, Denmark). CC corresponded to the determined mean values of at least two samples during the respective sampling month.

### 2.4. Statistical Analyses

Statistical analysis was performed using tailored software (JMP Pro, 17.0.0, 64-bit version, 2022 JMP Statistical Discovery LLC, Cary, NC, USA), and the results were considered significant at *p* < 0.05. BAPC spore counts per liter, SCC, and CC were log_10_ transformed to achieve values that are as normally distributed as possible. Selected farm management was merged before statistical analysis in order to reduce variables (see Supplementary Table S1). Linear mixed models were used to compare BAPC spore counts between “farms” and “sampling”. “Farm” and “sampling” and the simple interaction term served as fixed effects, and “cows per farm and sampling” served as a nested random effect. Tukey’s multiple comparisons for responses by condition were used (HSD all pairwise comparison with Tukey–Kramer adjustment) to analyze effects between farm and sampling. Pearson’s correlations were estimated, and the interpretation of effect size was based on Cohen [18], considering *r* = 0.10, *r* = 0.30, and *r* = 0.50 as small, medium, and large in magnitude, respectively. Additionally, the non-parametric Wilcoxon matched pairs test (using Bias for Windows, Version 11, Epsilon Verlag; Hochheim Darmstadt, Germany) was used to assess the teat cleaning efficacy based on the median BAPC spore count difference. A partition decision tree was generated using JMP Pro to gain insights into potential relationships of available data (including farm management characteristics) with obtained BAPC spore counts in PQM. For validation purposes and the definition of the optimal number of splits, the complete data set was split into a training and a test set. Figures were created using SPSS (IBM SPSS Statistics 27.0.1.0, 64-bit version, 2020 IMB Corporation, New York, NY, USA), and JMP Pro.

## 3. Results and Discussion

### 3.1. Teat Cleaning Efficacy

**Figure 2:**
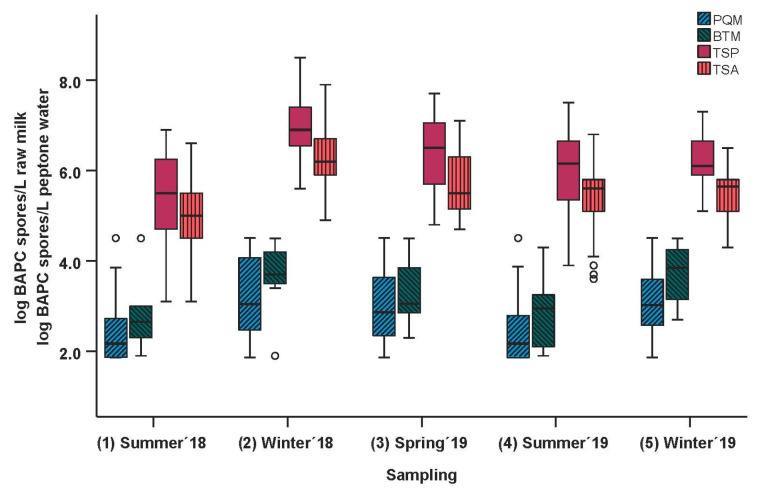
BAPC spore counts in pooled quarter milk samples (*n* = 631) and bulk tank milk samples (*n* = 40) (log BAPC spores/L raw milk) and log BAPC spores/L peptone water from teat swab samples obtained prior to (TSP; *n* = 191) and after (TSA; *n* = 191) teat cleaning. Each box-and-whisker plot represents aggregated data from farms A-H for each sampling. Black horizontal lines in the boxes indicate medians. Circles indicate outliers.

Aggregated data for each sampling season and sample material are illustrated as box-and-whisker plots in Figure 2. 

The BAPC spore counts of the teat swab samples collected in buffered peptone water prior to (TSP) and after (TSA) the routine teat cleaning by the farmer were analyzed. Results for TSP and TSA are illustrated as red boxes. Spore counts ranged from 3.4 to 8.4 log BAPC spores/L peptone water before cleaning and from 3.2 to 7.8 log BAPC spores/L after teat cleaning. The Wilcoxon matched pairs test showed significantly lower BAPC spore counts after teat cleaning (*p* < 0.0001), with a median difference of 0.6 log units between BAPC spore counts prior to (TSP) and after (TSA) teat cleaning.

However, as the analyzed sample area was not standardized and varied due to different teat sizes, our interpretation focuses on relative results rather than absolute values. On average, pre-milking teat cleaning resulted in a reduction of 76.2% in the average initial spore count (−0.6 log units) and is consistent with other studies. Vissers et al. found that a basic pretreatment of teats before milking with an average removal of about 75% of attached spores is enough to ensure counts below 3.0 log spores/L bulk tank milk when BAPC spore counts are below 3.0 log/g silage [6].

Using conventional manual cleaning, Melin [19] observed a 66.5% reduction in the total number of *Clostridium tyrobutyricum* spores, which had been applied by dipping teats in a standardized slurry. Stadhouders [20] also compared different cleaning methods and concluded that good udder treatment can reduce BAPC contamination levels by a factor of 10, although some very efficient cleaning methods may be too time-consuming.

**Figure 3:**
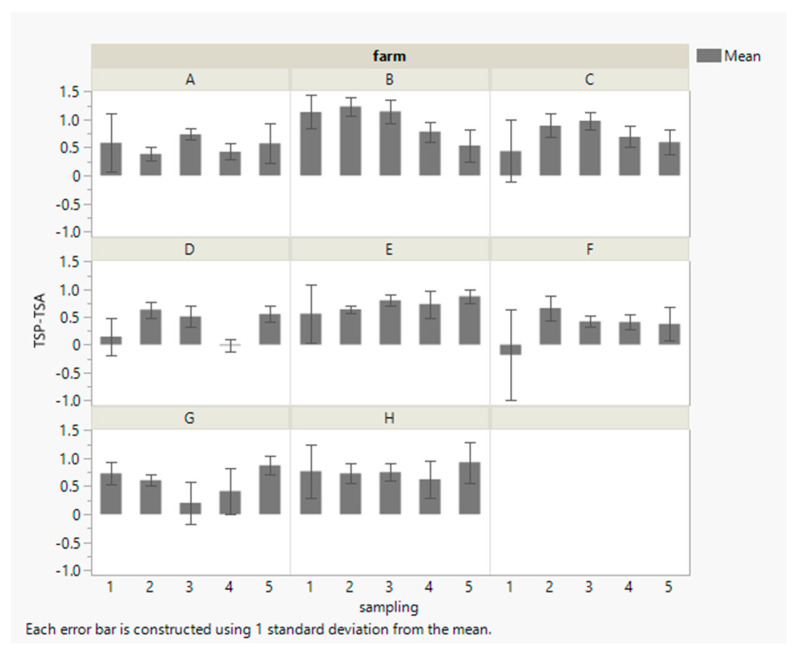
Arithmetic means (*n* = 4; except sampling 1: *n* = 8) of BAPC spore reduction (log BAPC spores/L) on teat skin after cleaning (teat skin sample prior to cleaning (TSP) compared to teat skin sample after cleaning (TSA)) on farms (**A**–**H**) throughout sampling seasons 1–5 (1: summer ’18, 2: winter ’18, 3: spring ’19, 4: summer ’19, 5: winter ’19).

Cleaning methods varied widely among the farms visited in this study. An overview of all cleaning methods used in this study is provided in Supplementary Table S1. The methods included dry paper towels (farms A and G), udder cloths (farms D, E, and H), wood wool (farm F), and a cleaning foam combined with a towel or cloths (farms B and C). Average spore reductions (log BAPC spores/L peptone water) and corresponding standard deviations per farm and sampling season are shown in Figure 3.

Farm B achieved the highest reduction in BAPC spores on teats with an average of 1.0 ± 0.3 log units (89%). This farm used a cleaning foam that was removed with a dry, disposable paper towel. A fresh paper towel was used for each cow. Magnusson et al. tested the effect of different teat cleaning methods in combination with different cleaning times on teats deliberately contaminated with *C. tyrobutyricum* spores [10]. They concluded that cleaning foam left on the teats for 30 s and then removed with a dry paper towel resulted in a similar spore reduction of 91% when compared to uncleaned teats without forestripping. In addition, they suggested the use of a damp, washable towel for maximum spore reduction.

Of all the farms, farms D and F achieved the lowest average reduction of 0.4 ± 0.2 and 0.3 ± 0.2 log units, respectively. The difference between farm B (highest reduction) and farms D and F (lowest reductions) was highly significant (*p* < 0.0001). Farm D varied the teat cleaning routine over the sampling seasons. Towels were used in sampling seasons 1, 2, and 4. A single towel was used to clean the teats of several cows. After milking, the towels were washed by hand and reused for several days. In general, Rasmussen et al. [21] attributed good cleaning effects to cotton towels in terms of bacterial spore reduction due to the fabric structure. However, the poor teat cleaning results of farm D were probably due to the use of a single towel for multiple animals and the subsequent contamination of the teats, rather than a significant reduction in spores. In sampling seasons 3 and 5, the first step of teat cleaning on farm D involved wet udder cloths. The second step was to dry the teats with dry udder cloths. A single cloth was used for a maximum of two animals before disposal. However, despite improvements in the cleaning routine in seasons 3 and 5, spore reduction on farm D remained poor, indicating the need for further improvements in teat cleaning and barn management. Interestingly, farm F was the only farm that used wood wool for teat cleaning. A new bundle of wood wool was used for each cow. The use of wood wool for teat cleaning has a long history. Ruf et al. found a reduced concentration of Gram-positive bacteria in raw milk derived from teats cleaned with wood wool compared to the milk from teats that were cleaned with normal udder cloths [22]. This could be the result of antimicrobial tannins naturally present in wood wool [23]. However, the ability of wood wool to reduce BAPC spores may warrant further investigation.

This study highlights the wide variation that can be observed in routine teat cleaning procedures. However, it is important to note that a lower reduction in spore counts cannot be attributed simply to the cleaning method. In fact, potential biases must be considered when interpreting cleaning effects. First, the variation in initial BAPC spore counts on teat skin from different farms must be considered: a significant positive strong correlation (*n* = 191, *p* < 0.0001, *r* = 0.91) was observed between the clostridial spore counts of the dirty and the clean teats. If the initial BAPC spore counts on the teats were high, counts remained high after routine teat cleaning, emphasizing the importance of keeping udders clean despite subsequent cleaning. Farms D and F, already discussed above as the farms with the lowest reduction in BAPC spore counts after cleaning among all farms, had low initial BAPC spore counts on teats before cleaning (farm D: 5.6 ± 1.9, farm F: 5.3 ± 1.6 log BAPC spores/L peptone water). Vissers et al. also concluded that little reduction can be gained from pretreatment of slightly contaminated cows [6].

In addition, seasonal variations may introduce bias. No significant differences between sampling seasons were observed for the reduction in BAPC spores on teat skin when aggregated data were compared (as indicated also by columns and corresponding SD shown in Figure 3). Therefore, the reasons for seasonal variations in BAPC spore counts in the dairy environment are discussed in more detail for the pooled quarter milk samples. However, the low average initial BAPC spore counts of farms D and F were the result of bias due to very low spore counts in sampling seasons 1 and 4 (summer) and high spore counts in sampling seasons 2, 3, and 5 (winter and spring). Due to very low initial BAPC spore counts on the teats in the summer samplings (1 and 4), almost no or a small reduction of 0.1 log units was achieved. In fact, farm F even showed a slight increase in the average spore count (+0.2 log units) after teat cleaning during the first summer sampling (1). Closer examination revealed that this increase was due to two of the eight samples. A similar increase in the spore count after teat cleaning was again observed for farm F in the second summer sampling (+0.02 log units, caused by one sample in sampling 4). The increase in BAPC spore counts can be explained by small variations in the teat swab sampling procedure among samplings and operators. Considering only the winter samples, farms F and D showed an average reduction of 0.6 ± 0.0 and 0.5 ± 0.1 log units, respectively, which is in line with the average cleaning effect achieved in this study.

In short, despite some variations described above, the overall average BAPC spore reduction of all farms was consistent, albeit with seasonal variations: 0.5 log units for samplings 1 and 4 (summer) and 0.7 log units for samplings 2, 3, and 5 (winter and spring), resulting in an overall average spore reduction of 0.6 ± 0.3 log units.

### 3.2. BAPC Spore Counts in Milk

#### 3.2.1. BAPC Spore Counts in PQM

**Figure 4:**
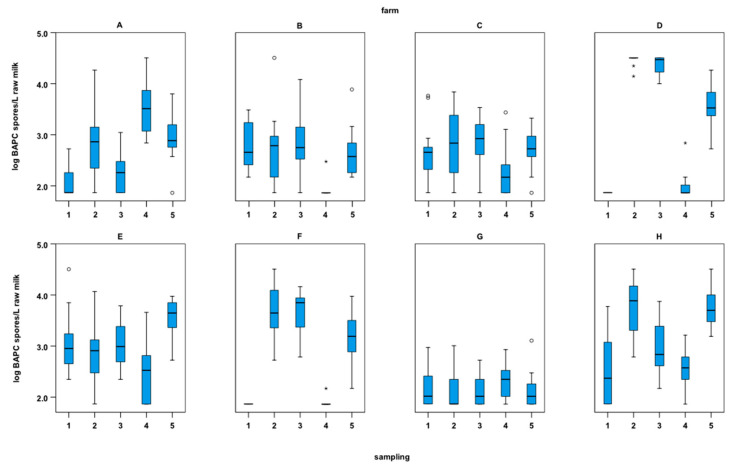
Box-and-whisker plots of BAPC spore counts in pooled quarter milk samples (log BAPC spores/L milk) from 16 cows (*n* = 16) collected per farm (**A**–**H**) and sampling (1: summer ’18, 2: winter ’18, 3: spring ’19, 4: summer ’19, 5: winter ’19). Circles indicate outliers and asterisks indicate extreme outliers.

BAPC spore counts in PQM samples ranged from ≤1.9 log to 4.5 log BAPC spores/L milk. The box-and-whisker plots in Figure 4 show aggregated BAPC spore counts per sampling and farm. 

Multiple comparisons showed significant differences in BAPC counts in PQM samples among some of the selected farms (*p* < 0.0001). These differences may at least partly be attributed to different management practices on the test farms. Thus, some farm-specific results and some aspects of farm management merit further discussion. An overview of the obtained farm management data is given in Supplementary Table S1.

High average spore counts were observed on farms D, E, F, and H. These farms had average BAPC spore counts ≥2.9 log/L PQM. Farms E and H both have a free stall and a tandem milking parlor without (farm E) and with (farm H) milking rank order. Interestingly, farm E changed the teat cleaning routine several times during the experiment, although no remarkable effects on BAPC spore counts were observed. In terms of farm management data, farms D and F have several similarities. They stand out among the test farms as they were the only tie-stall farms and used a pipe milking system. In addition, both farms, D and F, did not perform post-milking teat dipping or disinfect milking clusters after a cow had been milked and before the cluster was used for the next animal. Milking on farms D and F was done according to a milking rank order. Farms D and F kept few dairy cows. On average, farm D had 22 dairy cows and farm F had 19 dairy cows. One might assume a higher influence on spore counts by bedding material, feed, and feces and barn air in tie-stall barns as the animals use the same place for defecation, resting, feeding, and milking. However, other authors found no significant relationship between the housing system and the contamination of bulk tank milk with anaerobic spores [6] or observed significantly higher percentages of milk containing >100 clostridial spores/L milk from free stalls than from tie stalls [24]. A detailed assessment of the influence of tie-stall and free-stall housing on BAPC spore counts is not possible based on the present data set and is also of limited relevance as traditional tie-stall housing is becoming increasingly rare. Especially for farms D and F, seasonal variations became also apparent. BAPC spore counts were very low (close or below the limit of detection) during sampling seasons 1 and 4 (summer) and very high (close or above the upper limit of detection) during samplings 2, 3, and 5 (spring and winter) (Figure 4). The difference between summer grazing and indoor tie-stall housing plus silage feeding was probably one of the main reasons for the high variation in BAPC spore counts between summer and winter/spring.

In general, clostridial spore counts in raw milk are thought to be higher in winter due to silage feeding and the fact that cows spend more time in the barn, resulting in increased contact with each other and bedding material. Feligini et al. [25] also observed that the abundance of *C. tyrobutyricum* spores in bulk tank milk is lower in summer than in winter and spring. The present data confirmed the seasonal variation in BAPC spore counts as seen in Figure 3 for PQM and in Figure 2 across all sample matrices. Aggregated BAPC spore counts in PQM samples of all farms were significantly lower in summer than in winter and spring (*n* = 631, *p* < 0.0001). Furthermore, differences in BAPC spore counts in PQM samples were not significant between samplings 1 and 4 (both summer; *p* = 1.0000) and samplings 2 and 5 (both winter; *p* = 0.3616).

In the past, late blowing of cheese was almost exclusively associated with milk produced in the winter, as cows were out to pasture during the summer. Nowadays, silage is sometimes fed all year round and cows may spend more time indoors also during summer [6]. In fact, farm F was the only one of the eight selected farms that did not feed grass silage as the main feed component during all five sampling periods, but only during winter.

Farm G had the lowest average count of clostridial spores (2.2 ± 0.3 log BAPC spores/L PQM; highly significant difference in comparison to all other farms; *p* < 0.0001) in PQM samples and the lowest variability of spore counts across all sampling seasons (Figure 4). Farm G, the largest of the selected farms in terms of number of dairy cows, had an average of 50 Brown Swiss cows in a free stall and used a herringbone milking parlor. The cows were continuously fed grass silage as the main feed component. Corn silage and medick hay were also offered. Surprisingly, the cows were not milked according to a milking order, nor was there an extra cluster for waste milk from animals with health problems. Normally these two measures are part of good farm management practice [26]. However, post-milking teat dipping was performed and the milking clusters were disinfected (peracetic acid) after a cow was finished milking and before the cluster was used for the next animal (exception: no intermediate cluster disinfection in season 5). Interestingly, farm G had consistently low BAPC spore counts and no significant differences in BAPC spore counts between the seasons (*n* = 80, *p* = 0.2331). The data indicate that it is possible to prevent elevated BAPC spore counts during winter and maintain low BAPC spore counts throughout the seasons.

One of the unique features of this study is the large data set of BAPC spore counts in PQM, which provides insights into the variation in clostridial spore levels among individual animals. Our study showed a variation in BAPC spore counts in PQM samples ranging from ±0.0 to ±0.7 log spores/L among animals per farm and sampling, with an average variation of ±0.4 log spores/L. This is consistent with observations by Secchi et al. [27] who investigated factors of variation in milk metagenomic relative abundances and also observed some animal-based variation for *Clostridiales*.

A significant strong positive correlation between the clostridial spore counts of cleaned teats and the corresponding average spore counts of the pooled quarter milk was determined (*n* = 191, *p* < 0.0001, *r* = 0.78). A visualization of this correlation is also provided in Figure 6. These results indicate a strong dependence of the BAPC spore counts in raw milk samples on the spore counts on the teats, and once again emphasize the relevance of dirty teats as a reservoir for clostridia and the importance of teat cleaning to ensure milk quality.

#### 3.2.2. BAPC Spore Counts in BTM

BAPC spore counts in BTM ranged from <73 to >32,000 across all sampling seasons with an average count of 3.3 log spores/L milk. The Dutch dairy industry implemented a quality payment system, which encourages farmers to keep BAPC spore counts below 3.0 log spores/L because this threshold ensures counts of <1.0 log spores/L pasteurized cheese milk after bactofugation [3]. Only 42.5% of the BTM samples had BAPC spore counts below 3.0 log spores/L milk and 10% showed BAPC spore counts ≤73/L milk. Lower counts were found in a Dutch study by Vissers et al. [3]: about 66% of BTM samples showed a count of <3 log spores/L, and the average count was 2.7 log spores/L.

A significant strong positive correlation was observed between the average BAPC spore counts of the PQM samples from each farm for a given sampling season and the corresponding BTM samples (*n* = 40, *p* < 0.0001, *r* = 0.84). However, the average BAPC spore count of the BTM samples was significantly higher (average difference of 0.5 ± 0.2 log BAPC spores/L raw milk) than the average spore count of the PQM samples. A difference in BAPC spore counts between PQM and BTM samples from the same farm during the same sampling was observed especially at high BAPC counts. On farm G, the farm with the lowest average BAPC spore counts among all farms, BAPC spore counts in PQM and BTM samples were more similar (0.2 log unit difference). In addition, on farms B and H, the farms that showed the highest average BAPC spore reductions on teats after cleaning (Figure 3), the difference was lower (0.3 log difference on farm B and 0.0 on farm H). The results indicate that after PQM sampling, further contamination may occur during milking and storage. On the one hand, additional dirt including BAPC spores may be transferred from teat skin to milk during milking. On the other hand, barn air may contribute as an additional reservoir for clostridial contamination, although Stadhouders [20] and Martin et al. [8] considered barn air to be of limited relevance for milk contamination with BAPC spores. However, limited data are available on the extent of the contribution of barn air to BAPC counts in milk, and this topic may merit further investigation. Further contamination can also occur during passage through the milking equipment and in the milk tank. Another factor to consider in our study is the number of dairy cows contributing to the BTM samples: the BTM samples included not only the raw milk of the 16 individual cows selected for the PQM samples, but also the milk of all the remaining healthy and lactating cows on the farm at the given sampling time. Nevertheless, the strong correlations found in BAPC spore counts between PQM and BTM samples indicate that the analysis of BTM, as currently performed during routine testing, is a suitable indicator of BAPC spore contamination levels on dairy farms.

The seasonal influence on clostridial spore counts in BTM was not as clear as for PQM: there was no significant difference in the spore counts among all farms between the sampling seasons. We assume that the lack of a significant influence was due to the small sample size (*n* = 40). However, the spore counts in BTM were numerically lower in summer than in winter and spring (Figure 2).

### 3.3. Correlations of BAPC Spore Counts in BTM with Somatic Cell Count (SCC) and Colony Count (CC)

Among the parameters routinely tested to assess raw milk quality and determine the payment to farmers, the main parameters that provide some indication of the bacteriological status of milk are colony count and somatic cell count. Since BAPC spore count, SCC, and CC are all somehow dependent on the overall hygiene conditions in the barn, there is an ongoing debate as to whether there may be some correlation between CCs and SCCs and BAPC spore counts. For example, Barkema et al. [28] were able to associate certain farm management practices with low, medium, and high SCCs in bulk tank milk, which are likely to influence BAPC spore counts in the same way: some of the practices that result in low SCCs also are known to have a positive impact on LBD prevention, such as regularly clipping udder hair to reduce dirt adhesion [28,29].

However, in our study, no significant correlation was found between the average CC and the BAPC spore counts in BTM (*n* = 40, *p* = 0.1148, *r* = 0.2533). This may be partly explained by the fact that, unlike other microorganisms in their vegetative state, clostridia are present on teats and in milk as endospores. Furthermore, it is important to consider that the CC was based on the routinely acquired monthly average determined by the dairy and BAPC spore counts on the individual BTM sample analyzed in the current study.

There was also no significant correlation between the BAPC spore counts and SCCs of the bulk tank milk (*n* = 40, *p* = 0.5083, *r* = −0.1077). A notable difference between BAPC spore counts and SCCs is that elevated SCC levels, as an indicator of mastitis, can often be linked to specific dairy cows with elevated SCC due to animal health [30] and BAPC spore counts rarely seem to be associated with individual animals, but rather the overall hygienic status and degree of udder contamination of the herd. However, a limitation of this part of the study is the small number of BTM samples (*n* = 40), and further research is recommended.

A larger data set was available for PQM samples. Potential correlations at the individual cow level were therefore addressed by comparing SCCs and BAPC spore counts in PQM. Our results from BTM were confirmed as there was no significant correlation between the BAPC spore counts and SCCs of PQM (*n* = 629, *p* = 0.0675, *r* = −0.0730).

Despite some limitations discussed above, our results provide a first indication that CC and SCC, although also good indicators of barn hygiene, do not correlate with BAPC spore counts in milk.

### 3.4. Correlations of BAPC Spore Counts in PQM with Farm Management Data

Supplementary Table S1 provides detailed farm management data that were obtained in this study that may be of potential relevance to BAPC counts in milk. Farm management data collected through a survey include information on cow numbers, housing conditions, milking equipment and procedures, feeding strategies, and teat cleaning and dipping.

**Figure 5:**
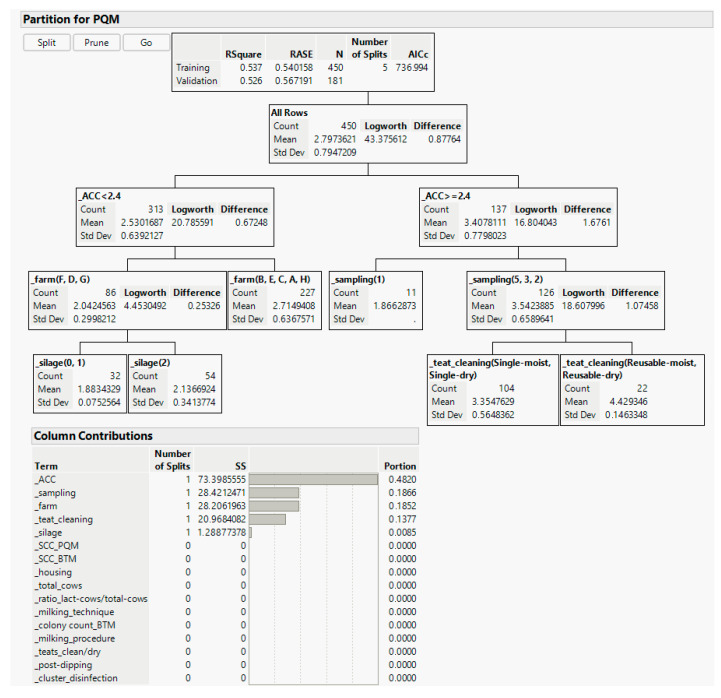
Validated partition decision tree based on the predictor (log BAPC spore counts per liter pooled quarter milk (PQM)) and response values (farm management data listed in “column contributions” and described in more detail in Supplementary Table S1.

To identify important relationships between BAPC spore counts in PQM samples and farm management data, we generated a partition decision tree, dividing our data according to a relationship between the predictor (BAPC spore counts in PQM) and the response values (farm management data shown in “column contributions” in Figure 5 and described in more detail in Supplementary Table S1). The resulting tree and respective column contributions are provided in Figure 5.

The first split of the partition decision tree divides our data set into samples with an average cow cleanliness (ACC) < 2.4 and an ACC ≥ 2.4. Samples with an ACC < 2.4 had mean BAPC spore counts of 2.5 ± 0.6 log/L PQM, whereas samples with an ACC ≥ 2.4 had mean BAPC spore counts of 3.4 ± 0.8 log/L PQM. The “column contribution” table in Figure 5 shows that the ACC provided the largest contribution to the partition tree with 48.2%, indicating the most relevant relationship in our data set between ACC and BAPC spore counts in PQM. The next splits based on the split candidates “sampling” and “farm” confirm the significant differences in our data set between farms and sampling seasons, which have been already discussed above. The column contributions of “sampling” and “farm” to the partition tree are 18.7% and 18.5%, respectively. Next, the data are partitioned by teat cleaning procedures (contributing with 13.8%). Figure 5 shows that the factor “single use” or “reusable” is more important than the category moist or dry, because samples where disposable cleaning was used resulted in lower BAPC spore counts in PQM (mean 3.4 log/L) than samples obtained from cows after teat cleaning with reusable towels (mean BAPC spore count in PQM: 4.4 log/L). The last split is based on the silage feed used, indicating that when no silage or one type of silage was fed, BAPC spore counts in PQM were lower than those when two types of silage were used. It is well known from previous studies that silage quality has a major influence on BAPC spore counts in milk [3]. However, the differences in silage feeding strategies among the selected farms in this study appeared to be of limited relevance, as also indicated by the column contribution of only 0.9%. All the other parameters of the obtained farm management data, including general parameters such as housing or number of cows and differences in milking procedures, such as milking rank order and post-milking teat dipping, showed no visible dependencies on BAPC spore counts in PQM in the current study (contributions of 0.0% to the partition decision tree). Furthermore, our findings that SCC and CC did not correlate with BAPC spore counts in milk were confirmed, as can be seen from the 0.0% column contributions. However, it must be also considered that there are factors that were not addressed in the current study and, furthermore, that there are factors that are beyond the control of the farmer, such as weather conditions and soil contamination [6].

To validate the statistical analysis based on the partition decision tree, we divided our data set into a training set and validation test set. Similar R-squared values between the training (0.537) and validation (0.526) data sets (see the top of the decision tree in Figure 5) confirm that the shown partition tree adequately represents our data set and indicates potential relationships. Thus, the resulting most important relationship between ACC and BAPC spore counts in PQM samples is discussed in more detail below.

### 3.5. Correlations of BAPC Spore Counts in Raw Milk with the Average Cow Cleanliness

Average cow cleanliness according to the hygiene scoring of Cook and Reinemann [13] is a useful tool for mastitis risk management focusing on teat contamination with manure. Nadeau et al. [12] used a similar scoring scheme according to Schreiner and Ruegg [11] and concluded that cow cleanliness was worse in herds with high spore counts than in herds with low spore counts. Zucali, Bava, Colombini, Brasca, Decimo, Morandi, Tamburini, and Crovetto [7] further emphasized the importance of udder hygiene during the summer, as they observed that when the herd contained more than 40% dirty dairy cows, the clostridial spore count in raw milk increased by 15% [11]. Martin et al. [8] also identified udder hygiene as an important factor that affects BAPC spore levels in milk. An improved hygiene score for mastitis risk assessment (named ACC in this study), which takes into account different manure transfer mechanisms, was proposed by Cook and Reinemann [13]. They suggested four possibilities for manure transfer to the udder and teats: (1) direct transfer by lying down, (2) transfer via the legs during lying or contact between dirty legs and udder, (3) splash transfer during walking through manure slurry, and (4) transfer via a dirty tail. Since the transfer of clostridial spores into raw milk occurs mainly via dirty teats, we hypothesized that this scoring system might be a potential tool to estimate BAPC contamination within a herd and thus provide a rough indicator of cheese milk quality.

**Figure 6:**
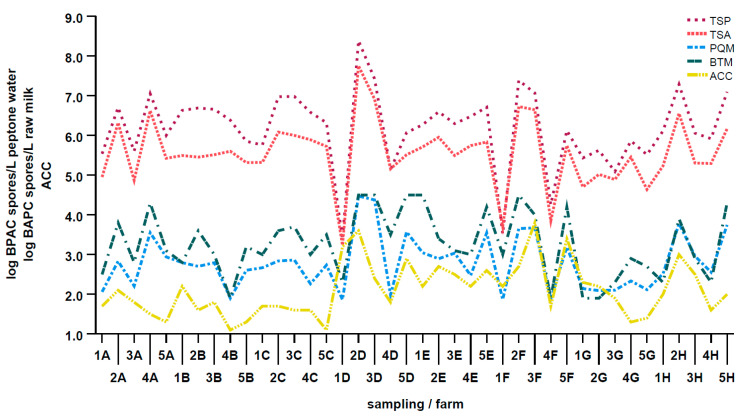
Visual summary of the obtained results and correlations. BAPC spore counts in pooled quarter milk samples (PQM) and BTM (bulk tank milk) in log BAPC spores/L raw milk, in buffered peptone water obtained from swabs after sampling teat skin prior to (TSP) and after (TSA) cleaning (in log BAPC spores/L peptone water), and average cow cleanliness (ACC) (scores 1–4 according to Cook and Reinemann (2007)). Samples were collected from eight dairy farms (A-H) during sampling seasons 1–5 and were aggregated to one data point for each farm visit (*n* = 40).

We observed a strong positive correlation between the average BAPC spore counts in PQM and the corresponding ACC values (*p* = 0.0007, *r* = 0.5142). Additionally, the correlation between ACC and the BAPC spore counts in BTM was determined: the correlation coefficient *r* was lower than that for the PQM samples, indicating a moderately positive but still significant correlation (*p* = 0.0091, *r* = 0.4071). In summary, Figure 6 provides a visualization of important correlations found in this study, including similarities between ACC, PQM, BTM, TSP, and TSA data. Our results suggest that the ACC, although developed as a tool for mastitis risk assessment, may be a useful tool that could be implemented by farmers or farm advisors to provide a rough estimate of BAPC spore contamination in raw milk.

## 4. Conclusions

The present study highlights the importance of pre-milking teat cleaning as a necessary measure to reduce the risk of late blowing in cheese by reducing clostridial spore counts in raw milk. On average, routine pre-milking teat cleaning resulted in a reduction of 0.6 units, which equals a reduction of 76.2%. A strong positive correlation was found between BAPC spore counts of cleaned teat skin and the corresponding pooled quarter milk samples. In addition, a strong positive correlation was observed between BAPC spore counts in pooled quarter milk and bulk tank milk, indicating the route of transmission of clostridial spores to raw milk via dirty teats during milking. Significant differences in BAPC spore counts between selected farms were identified and potential influencing factors and farm management decisions were discussed. In agreement with other studies, BAPC spore counts were lower in summer than in winter in all sample matrices. No significant correlations were observed between BAPC spore counts and colony counts or somatic cell counts in milk. However, the average cow cleanliness scoring system, although developed as a mastitis risk assessment tool, showed a strong correlation with BAPC spore counts. Thus, the average cow cleanliness scoring system may be a practical tool that could be used by farmers and farm advisors for a quick and rough assessment of udder hygiene and subsequent potential contamination of raw milk with clostridial spores. Finally, the results of this study highlight that hygiene measures and farm management play an important role in ensuring raw milk quality and preventing cheese spoilage by butyric acid producing clostridia.

## Data Availability

The data presented in this study are available upon reasonable request from the corresponding author (J.B.). The data are not publicly available due to privacy restrictions.

## Supplementary Materials

The following supporting information can be downloaded at: https://www.mdpi.com/article/10.3390/microorganisms11051337/s1, Table S1: Overview of farm management data collected from farms A–H during sampling seasons 1–5.
