# Supplementary material for: Importance of Pre-Milking Udder Hygiene to Reduce Transfer of Clostridial Spores from Teat Skin to Raw Milk
Source: Microorganisms. 2023 May 19;11(5):1337. doi: 10.3390/microorganisms11051337 (PMC10221102; doi:10.3390/microorganisms11051337)
Supplement: Supplementary file 1 [file microorganisms-11-01337-s001.zip › microorganisms-2337619-supplementary.pdf]

Farm	A	B	C	D	E	F	G	H
**Total number of dairy cows (number of lactating cows)**[1]	23 (21)	28 (23)	40 (34)	22 (20)	30 (25)	19 (16)	50 (44)	18 (16)
**Main cow breed**	HF	SI	BS	SI	HF	SI	BS	SI
**Milk yield/cow [kg]**[1]	25.7	25.6	26.7	16.6	25.3	21.0	30.8	31.3
**Housing and milking technique**	Free stall<br>Herringbone milking parlor (MP)	Free stall<br>Tandem MP	Free stall<br>Tandem MP	Tie stall<br>Pipe milking	Free stall<br>Tandem MP	Tie stall<br>Pipe milking	Free stall<br>Herringbone MP	Free stall<br>Tandem MP
**Main feed**[2]	Grass silage	Grass silage	Clover grass silage	Grass silage	Grass silage	Grass silage (2, 5)<br>Pasture grass (1, 3, 4)	Grass silage	Grass silage
**Additional feed**[2]	Corn silage (1, 3, 5)<br>Pasture grass (1, 3, 4)<br>Hay (2, 5)	Corn silage (1, 2, 5)<br>Pasture grass (1, 4)<br>Hay (2, 3, 5)	Corn silage (2-5)<br>Pasture grass (4)	Pasture grass (1, 4)<br>Hay (1-5)	Corn silage (1-3, 5)<br>Hay (1-5)	Hay	Corn silage<br>Alfalfa hay	Corn silage (1-5)<br>Pasture grass (1, 4)<br>Hay (1-5)

**Milking rank order**[3]	✓	×	×	✓	×	✓	×	×
**Separate milking cluster for waste milk from animals with health issues**[3]	×	✓	✓	✓	×	✓	×	✓
**Teat cleaning**	Dry paper towel (single use, one item per cow)	Cleaning foam + dry paper towel (single use, one item per cow)	Cleaning foam + dry udder cloth (single use, one item per cow)	- Udder towel (1, 2, 4; reusable, one item for several cows) - Udder cloth (3, 5; single use, one item for max. two cows)	- Sponge cloth in disinfectant (1; reusable, one cloth for all cows) - Moist udder cloth (2, 3, 5; single use, one item per cow) - Dry udder cloth (4; single use, one item per cow)	Wood wool (single use, one item per cow)	Dry paper towel (single use, one item per cow)	Moist udder cloth + dry udder cloth (single use, one item per cow)
**Post-milking teat dipping**[4]	✓	✓	✓	×	✓	×	✓	✓

**Teat check before cluster attachment (clean & dry)**	✓	✓	✓	×	×	✓	×	✓
**Milking cluster disinfection prior attachment to next cow**	× (1-4)<br>✓ (5, only after cows with health issues)	×	×	×	× (1)<br>✓ (2-5, only after cows with health issues)	×	✓ (1-4)<br>× (5)	✓

[1] average from 5 samplings
[2] For statistical analysis "silage" was aggregated for each sampling and farm: 0 = no silage was fed; 1 = one silage type was fed; 2 = two different silage types were fed
[3] For statistical analysis data on "milking procedure" was aggregated for each sampling and farm: 00 = no rank order and no extra cluster; 01 = no rank order but an extra cluster was used; 10 = rank order but no extra cluster was used; 11 = rank order plus extra cluster was used
[4] for statistical analysis data on post-milking dipping (post-dipping) was aggregated for each sampling and farm: 0 = no post-dipping was performed; 1 = the used dipping agent was based on lactic acid; 2 = the used dipping agent was based on iodine
(1, 2, 3, 4, 5) = Sampling on which this information applies; (1: summer '18, 2: winter '18, 3: spring '19, 4: summer '19, 5: winter'19)

✓ = yes
× = no
HF = Holstein-Friesian, BS = Brown Swiss, SI = Simmental
